# Supplementary material for: Pawsitively sad: pet-owners are more sensitive to negative emotion in animal distress vocalizations
Source: R Soc Open Sci. 2019 Aug 21;6(8):181555. doi: 10.1098/rsos.181555 (PMC6731714; doi:10.1098/rsos.181555)
Supplement: Stimulus check examining an independent sample's categorisation of the animal sounds. Supplementary details on the adult, infant crying sounds. [file rsos181555supp1.docx]

**Supplementary Information**

**Pawsitively sad: Pet-owners are more sensitive to negative emotion in animal distress vocalisations**

Christine E. Parsons^1^, Richard T. LeBeau^2^, Morten L. Kringelbach^3^, Katherine S. Young^2,4^

**Stimulus check**

Prior to running the main experiment, we did not obtain information on the age of the animals vocalising or the context in which the vocalisation was produced (the ‘reason’ for the vocalisation). To address this limitation, we carried out a short post-experiment stimulus check to investigate whether the perceived age or context differed between the cat and dog vocalisations.

**Participants**

Thirty-one participants performed an online categorisation task, using the survey platform Prolific. Inclusion criteria were no hearing difficulties, and aged between 18 and 60 years. Eight participants reported being cat-owners, four were dog-owners, two owned both and seventeen owned neither. Six reported being cat-lovers, 11 were dog-lovers, and 13 said they loved both equally, and one participant said they were neither a cat nor dog lover.a

**Task**

Participants were asked to listen to the 15 dog and 15 cat sounds once, and categorise the sound as ‘cat’ or ‘kitten’, ‘puppy’ or ‘dog’ as relevant, and as either ‘hungry’, ‘in pain’, ‘seeking general attention’ or if they ‘cannot say’. The sound order was randomised across participants, and the order of these two questions was also randomised. The task also contained an attention check, where participants were asked to select if a meow or a purr had been played. All participants passed this attention check. The survey task took on average 5.40 mins to complete (SD= 2.22mins).

**Results**

*Adult or infant animal categorisation*

As presented in Figure 1a, all cat stimuli were categorised with high proportions of participants agreeing on the cat or kitten selection (all >70% agreement). Four of the cat stimuli were consistently perceived as kittens and eleven were perceived as adult cats. For the dogs, (Figure 1b), there was much greater variation in whether people perceived it as a puppy or dog (ranging from 50% to 80% agreement). Eight of the dog stimuli were perceived as puppies (>50%) and seven were perceived as adult dogs. However, a Chi Square test indicated no significant difference overall in the distribution of choices for cats vs. dogs (Figure 1c; Chi Square test= 0.10; p=0.76).

Figure S1. a. For each cat stimulus (c01 = cat 1), we present the percentages of times it was chosen as a ‘cat’ or ‘kitten’. b. For each dog stimulus (d01= dog 1), we present the percentages of times it was chosen as a ‘dog’ or ‘puppy’. c. Averaging across all stimuli, we present the proportion of times where ‘adult’ or '’infant' was chosen for the cats and dogs.

*The ‘reason’ for the vocalisation*

The most commonly selected reason for the vocalisation was ‘seeking general attention’ across both the cats (SFigure 2a) and dogs (SFigure 2b). Some stimuli were more clearly selected as ‘pain’ (e.g., cat 09, dog 04), but in general, there was a lot of variability in the category selected for each stimulus. Like for the age categorisation, we found no difference in distribution of choices selected in this task for cats vs. dogs (Chi Square test= 2.95; p=0.40)

Figure S2. a. For each cat stimulus (c01 = cat 1), we present the percentages of times each ‘reason’ was chosen. b. For each dog stimulus (d01= dog 1), we present the percentages of times each ‘reason’ was chosen. c. Averaging across all stimuli, we present the proportion of times where each reason was chosen for the cat and dog stimuli respectively.

*Physical parameters of four categories of sounds from the OxVoc Database*

S. Table 1 presents the estimated pitch of the cat, dog, adult and infant sounds included in the OxVoc Database, where 15 sounds were used from each category. Overall, adult sounds had the lowest pitch.

S. Table 1. Estimated Pitch of the four sound categories (N=60), with SD

|  | N | Mean Calculated Fundamental Frequency | SD |
| --- | --- | --- | --- |
| Cat meows | 15 | 406.98 | 126.88 |
| Dog whines | 15 | 471.58 | 54.93 |
| Infant cry sounds | 15 | 472.29 | 80.46 |
| Adult cry sounds | 15 | 339.84 | 64.34 |
| Total | 60 | 422.67 | 100.48 |

*No differences by pet ownership in the perception of human crying sounds*

We also compared the four participant groups (no pet, dog only, cat only and cat and dog) on their responses to infant and adult crying (see S.Fig 3). There were no significant differences between the groups (infant crying: F(3, 539)=0.04, p=0.99; adult crying: F(3, 539)=0.21, p=0.89);

S. Figure 3. Pirate plots presenting valence ratings from the four participant groups for infant crying (top panel) and adult crying (bottom panel). Raw data is represented by the black dots, the vertical bar represents the mean, the coloured regions show smoothed densities and the rectangle shows the confidence intervals.
